# The optoelectronic role of chlorine in CH_3_NH_3_PbI_3_(Cl)-based perovskite solar cells

**DOI:** 10.1038/ncomms8269

**Published:** 2015-06-12

**Authors:** Qi Chen, Huanping Zhou, Yihao Fang, Adam Z. Stieg, Tze-Bin Song, Hsin-Hua Wang, Xiaobao Xu, Yongsheng Liu, Shirong Lu, Jingbi You, Pengyu Sun, Jeff McKay, Mark S. Goorsky, Yang Yang

**Affiliations:** 1 Department of Materials Science and Engineering, University of California, Los Angeles, California 90095, USA; 2 California NanoSystems Institute, University of California, Los Angeles, California 90095, USA; 3 WPI Center for Materials Nanoarchitectonics (MANA), National Institute for Materials Science, Tsukuba 305-044, Japan

## Abstract

Perovskite photovoltaics offer a compelling combination of extremely low-cost, ease of processing and high device performance. The optoelectronic properties of the prototypical CH_3_NH_3_PbI_3_ can be further adjusted by introducing other extrinsic ions. Specifically, chlorine incorporation has been shown to affect the morphological development of perovksite films, which results in improved optoelectronic characteristics for high efficiency. However, it requires a deep understanding to the role of extrinsic halide, especially in the absence of unpredictable morphological influence during film growth. Here we report an effective strategy to investigate the role of the extrinsic ion in the context of optoelectronic properties, in which the morphological factors that closely correlate to device performance are mostly decoupled. The chlorine incorporation is found to mainly improve the carrier transport across the heterojunction interfaces, rather than within the perovskite crystals. Further optimization according this protocol leads to solar cells achieving power conversion efficiency of 17.91%.

## Abstract (web summary)

Chlorine incorporation into CH_3_NH_3_PbI_3_ improves solar cell performance, but its optoelectronic role is still unclear. Here the authors present a strategy that decouples the morphological impact, to reveal that chlorine incorporation affects carrier transport across the heterojunction interface rather than within the perovskite crystal.

## Graphical abstract

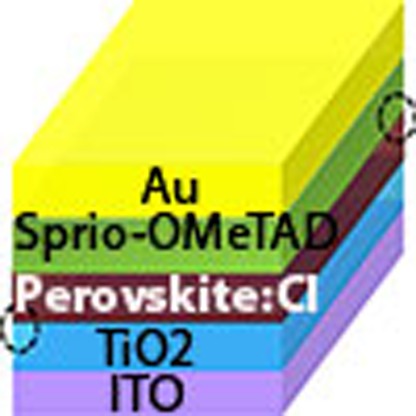

Hybrid perovskite materials, particularly CH_3_NH_3_PbX_3_ (X=Cl, Br, I), possess the advantages of an ideal absorber—appropriate and adjustable bandgap, high absorption coefficient, long carrier diffusion length and high tolerance of chemical defects^1^,^2^,^3^,^4^,^5^,^6^. By capitalizing on the techniques of thin-film electronics, perovskite solar cells have achieved power conversion efficiencies (PCE) rapidly approaching 20% (refs 7, 8, 9, 10, 11, 12). Moreover, due to its extremely low cost and scalable processibility, perovskites are regarded as one of the most promising photovoltaic (PV) materials that is potential to compete or integrate with crystalline silicon PVs. Substantial effort has been devoted to exploring the origins of the unique property of perovskite materials as well as advanced techniques for fabricating high performance devices. However, it remains challenging for the perovskite PV community to achieve a deep understanding of its composition, crystal structure and defect-associated optoelectronic properties for further device performance improvements.

Perovskites share the chemical formula of ABX_3_, where each ion follows the tolerance factor rule to occupy corresponding sites and serves as an independent building block. Numerous combinations of elements and molecules are thus available to create a diverse family of perovskites with distinct properties. Regarding **A** site cations, formamidinium (FA) has been shown to modify Pb(B)–I(X) bonding length and/or corresponding bond angles^13^,^14^,^15^,^16^,^17^. The resulting hybrid perovskites exhibit an altered band structure that extends its absorption edge to 850 nm. Similarly, materials property evolution has been documented when replacing lead by tin in **B** site, where the absorption edge can be successfully red-shifted to over 1,000 nm in tin-doped perovskites^18^,^19^,^20^,^21^. Exploiting possible **X** site occupants has received further attention^22^,^23^,^24^,^25^,^26^,^27^,^28^,^29^,^30^,^31^,^32^,^33^,^34^,^35^,^36^,^37^. Particularly, CH_3_NH_3_PbI_3−*x*_Cl_*x*_ has been reported to exhibit a substantially longer carrier diffusion length of over 1 μm compared with that of its counterpart CH_3_NH_3_PbI_3_ (∼100 nm), and device performance has consequently been elevated dramatically^38^,^39^. Through variance of the corresponding constituents, optoelectronic material properties can be manipulated, as they are largely determined by the crystal structure, the chemical potential and the defect property.

CH_3_NH_3_PbI_3−*x*_Cl_*x*_ has been serving as a typical system to examine the role of extrinsic ions, from the point of view of both crystallography and film morphology. Chlorine incorporation into the perovskite crystal structure was initially investigated^33^,^34^,^40^. Theoretically, a continuous solid phase of CH_3_NH_3_PbI_3−*x*_Cl_*x*_ should not form at high chlorine concentration, due to the large difference between the ionic radii of Cl and I. Experimental evidence for the presence of chloride in the perovskite films, regardless of the fabrication method, is lacking via the prevailing characterization techniques, a fact which indicates that the effects of Cl incorporation are beyond the scope of simply crystallographic considerations. Later, chlorine incorporation was investigated in the context of perovskites film growth to reveal that the improved film properties lead to the enhancement of device performance^28^,^30^,^31^,^35^. It has been suggested that crystallization begins from the nucleation of complex ion aggregates due to the limited solubility of the chlorine containing precursors, for example, PbCl_2_ in dimethylformamide. Rearrangement of constituent ions occurred during the subsequent crystal growth, where chlorine facilitates the release of excess organic component. It ultimately determines the crystallographic textures and grain structures of the perovskites. The accumulative efforts provide direct evidence that chloride inclusion governs the morphology evolution in the absorber, and consequently affect materials property and device performance.

Despite extensive inquiry, the effect of extrinsic ion incorporation is not yet fully understood, especially in the context of material properties that is irrelevant to morphological development. It is typically challenging to separate the origin of device performance enhancement from germane perovksite film evolution. Therefore, it is of great interest to establish a general platform that dissociates material properties from morphological effects due to incorporation of extrinsic ions. Here we adopt techniques that produce perovskite films in comparable film conformity with/without chlorine incorporation, attempting to decouple the morphology impact from device performance for the first time. The most influential morphological factors, in the context of surface coverage, film conformity and crystal structure, which are well known to affect device performance, have been largely alleviated, as characterized by X-ray diffraction (XRD), scanning electron microscopy (SEM) and X-ray photoelectron spectroscopy (XPS). Measurements based on time-resolved photoluminescence (TRPL), Kelvin-probe force microscopy (KPFM), transient photovoltage decay and capacitance–voltage (CV) indicate that the chlorine incorporation affects carrier transport across heterojunction interfaces rather than within the perovskite crystals. These results indicate a tremendous opportunity for rational incorporation of extrinsic elements in perovskites as desired, which facilitates their implementation for practical use in next-generation PV devices.

## Results

### An effective approach to decouple morphologic impact

**Figure 1:**
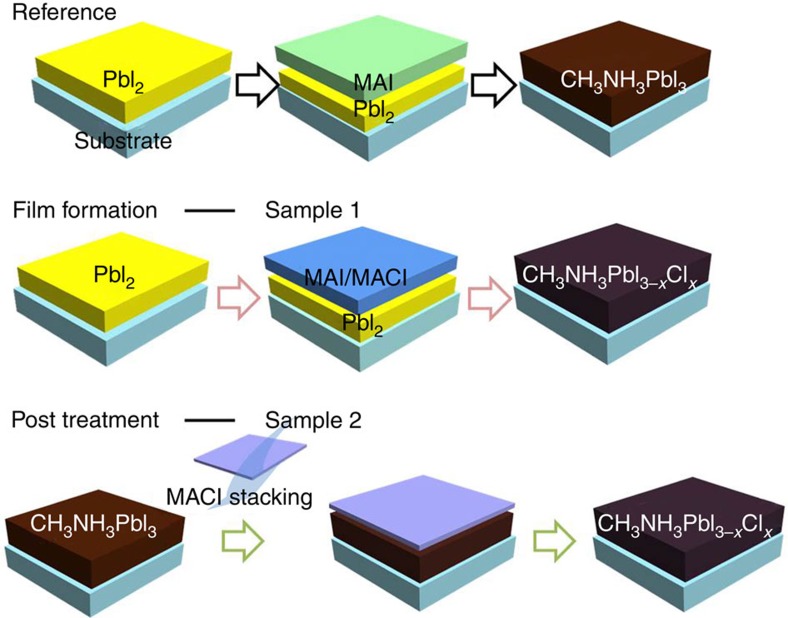
Perovskite film fabrication. Schematic illustration of different approaches to incorporate chlorine in the perovskite films. The Reference sample was obtained by a modified two-step solution process, where PbI_2_ and CH_3_NH_3_I were sequentially deposited via spin-coating and subsequently annealed. Sample 1 was obtained via the same procedure, except that a solution of CH_3_NH_3_Cl and CH_3_NH_3_I (for example, 1:10 in weight) was used instead of CH_3_NH_3_I solution to introduce chlorine during the film growth. Sample 2 was obtained by a proper treatment on the Reference sample with CH_3_NH_3_Cl solution.

Figure 1 schematically illustrates the rational approach to incorporating chlorine into the CH_3_NH_3_PbI_3_ film, where the CH_3_NH_3_Cl was intentionally involved either before or after the perovskite film formation (see Supplementary Methods). A CH_3_NH_3_PbI_3_ film without chlorine (Reference) was prepared by a modified two-step solution process^41^,^42^, where PbI_2_ and CH_3_NH_3_I were sequentially deposited via spin-coating and subsequently annealed. The as-formed CH_3_NH_3_PbI_3_ film possesses compact polycrystalline texture with full surface coverage and large grain size, features which are distinct from the poor film quality obtained from conventional one-step preparations. Sample 1 was obtained via the same procedure, except that a solution of CH_3_NH_3_Cl and CH_3_NH_3_I (1:10 in weight) was used instead of CH_3_NH_3_I solution to introduce chlorine during the film growth. In contrast, the Reference sample was further treated with a CH_3_NH_3_Cl solution to obtain Sample 2, where chlorine was most likely incorporated at the surface or grain boundaries of the CH_3_NH_3_PbI_3_ film. Thus, extrinsic chlorine in both Samples 1 and 2 was rationally introduced into the perovskite films in a controllable manner without severe interference of film conformity. While the platform established here uses chlorine as an example, it can be generalized for further investigation of the effects of other extrinsic ions.

### Characterization

**Figure 2:**
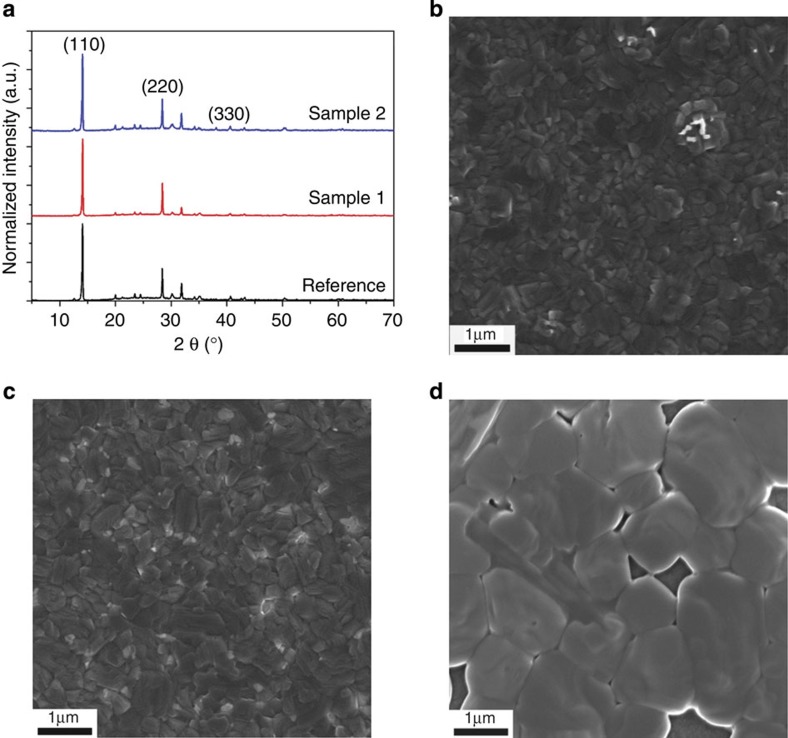
Phase and morphology characterization of perovskite film. (**a**) XRD patterns corresponding to perovskite films fabricated with different approaches (from bottom to up: Reference, Samples 1 and 2). All three samples show similar crystal structure. (**b**–**d**) Top-view SEM images of perovskite films of Reference (**b**), Sample 1 (**c**) and Sample 2 (**d**). Reference and Sample 1 exhibit similar morphology in terms of polycrystalline texture and grain sizes, while Sample 2 presents a distinct film morphology when compared with Reference and Sample 1.

XRD was conducted to characterize the crystal structure of each perovskite sample prepared on the TiO_2_-coated indium tin oxide (ITO) substrates (Fig. 2a). The perovskite films were prepared following the procedure that produces the optimized working device. It is found that both Samples 1 and 2 show similar crystal structures with distinctive (110), (220), (330) diffraction peaks centred at 14.2°, 28.3° and 42.9°, respectively. These peaks are in accordance with the major phases of CH_3_NH_3_PbI_3_, as shown in the Reference, regardless of the method of chlorine incorporation. However, Sample 1 showed slight difference in the diffraction profiles to that of the Reference, where the main peak slightly shifted to a higher degree. By assuming a tetragonal crystal structure in the perovskite, the calculated lattice parameter show ∼0.5% difference of the unit cell volume with respect to the Reference. This observation agrees with a previous claim that a CH_3_NH_3_PbI_3_ structure with a considerably low level of Cl doping is reasonable^40^. X-ray photoluminescence spectroscopy (XPS) was used to further examine the presence of Cl in the film (Supplementary Fig. 1). The resulting spectra revealed no detectable Cl signal in Sample 1 and the presence of a weak signal in Sample 2, results that are in agreement with recent characterizations of Cl in CH_3_NH_3_PbI_3−*x*_Cl_*x*_ films^28^,^30^,^33^. The rather small changes in lattice constant and scarcely detectable content of Cl clearly suggest that the incorporation of chlorine has negligible impact on the original crystal structure. Moreover, both Samples 1 and 2 exhibited the same diffraction intensity ratio for peaks, for example, (220)/(110), suggesting the similar crystal structure as compared to Reference. It indicates final products possess the same final phase ratios and domain orientation in the polycrystalline films, no matter what crystal growth/reorganization route has taken due to chlorine inclusion. The average crystal size for each sample, calculated according to the Scherrer's equation, was 45.7, 56.3 and 65.4 nm for the Reference, Sample 1 and Sample 2, respectively. In view of our result and previous work^5^, it would be carefully suggested to use CH_3_NH_3_PbI_3_(Cl) to denote the chemical formula of Samples 1 and 2 in this manuscript for more precise description of chemical composition and crystal structure.

The morphological evolution of perovskite films using various chlorine incorporation methods is shown in SEM images (Fig. 2). The Reference film exhibits uniform polygon grains of several hundred nanometres, which are comparatively larger than the results provided by XRD. Interestingly, some sporadic islands located at the adjunction of neighbouring grains are observed. It is notable that the CH_3_NH_3_PbI_3_ do form continuous and conformal films in the absence of incorporated Cl by taking the advantage of the pre-existed PbI_2_ framework, a feature quite different from films obtained from previously reported one-step deposition^33^,^38^. According to previous studies, the PbI_2_ framework could provide a sufficient number of nucleation sites to facilitate the growth of CH_3_NH_3_PbI_3_ on top. When chlorine was introduced during film growth in Sample 1 (Fig. 2c), the film exhibits similar polycrystalline texture and comparable grain sizes to the Reference sample, but no obvious islands are observed in Sample 1. The disappearance of islands is likely associated with enhanced film reconstruction and mass transport due to chlorine incorporation, where excess CH_3_NH_3_Cl easily escaped during the annealing process^30^. From the cross-sectional SEM images (Supplementary Fig. 2), Sample 1 shows a similar crystal texture to that of Reference with well-defined grains across the entire film thickness. In contrast, introduction of chlorine after film formation in Sample 2 produces a distinct morphology, as shown in Fig. 2d. Large voids in the vicinity of grains are frequently observed as well as grains with the size of up to 1 μm. An obvious discrepancy between the grain size measured by SEM and those calculated by XRD is observed due to limited instrumental resolution of the latter. The increased crystal size in Sample 2 may originate from an intensive crystal dissolve-recrystallization process promoted by the CH_3_NH_3_Cl solution. The limited duration of the Cl incorporation process suggests that a very quick dissolve-recrystallization process affects the evolution of film morphology and indicates a strong interaction between organic and inorganic species used to form the hybrid perovskite. Nevertheless, the perovskite films in Reference and Sample 1 exhibit similar crystal texture and film conformity, which can be further used to study their optoelectronic properties.

### Device performance based on Cl incorporation

**Figure 3:**
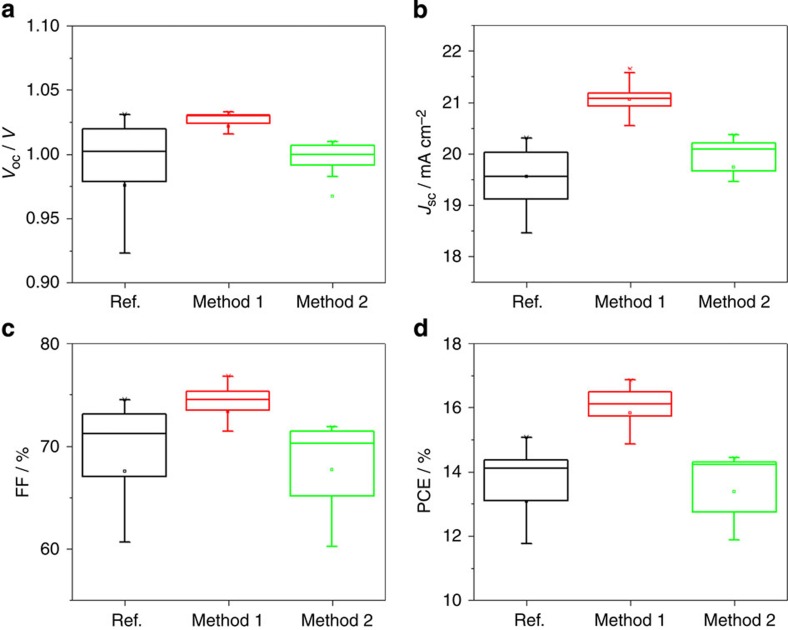
Device performance. PV parameters, for example, *V*_OC_ (**a**), *J*_SC_ (**b**), FF (**c**) and PCE (**d**), extracted from current–voltage measurements (under AM 1.5 radiation at ambient condition) of solar cells based on Reference and Samples 1 and 2. In comparison with devices based on Reference sample, the devices based on Sample 1 or Sample 2 shows superior or comparable performance, respectively. Ref., reference.

To correlate the incorporation of Cl in CH_3_NH_3_PbI_3_(Cl) films to device performance, we employed the above-mentioned absorber layers in a complete PV device that adopts the typical configuration of ITO/TiO_2_/Perovskite/Spiro-OMeTAD/Au. (see Supplementary Information) More than 10 nominally identical devices have been fabricated for each group of Reference, Sample 1 and Sample 2, respectively. Device performance is characterized by current density (*J*)–voltage(*V*) measurements under simulated AM 1.5 G (100 mW cm^−2^) solar irradiation in ambient conditions, with statistical distribution shown in Fig. 3 and the mean values summarized in Table 1. Reference devices produce open circuit voltages (*V*_OC_) in the range of 0.94–1.03 V, short circuit currents (*J*_SC_) in the range of 18.53–20.31 mA cm^−2^, fill factors (FF) in the range of 60.67–74.05% and the resulting PCE ranging from 11.77 to 15.08%. In comparison, superior performance is observed for devices based on Sample 1—*V*_OC_ in the range of 1.01–1.03 V, *J*_SC_ in the range of 20.26–21.66 mA cm^−2^, FF in the range of 69.7–76.8% and the resulting PCE ranging from 14.68 to 16.85%. On the other hand, devices based on Sample 2 show comparable performance to that of the Reference. The improved performance of devices based on Sample 1 reveals a positive effect arising from appropriate Cl incorporation in the CH_3_NH_3_PbI_3_(Cl) film. It can also be deduced that the relatively inferior performance of Sample 2, as compared with Sample 1, may be associated to the presence of the voids within the perovskite film provides shunting paths which deteriorate the device performance. It should be noted that both Sample 1 and the Reference sample exhibit similar conformity: continuous and void-free across the entire film but produce apparent differences in device performance. The enhanced performance of devices using Sample 1 in terms of *J*_sc_, *V*_oc_ and FF are possibly associated with the reduction of both parasitic current loss and series resistance. Compared with the Reference, it indicates that the improved device performance is less relative to the film morphology.

**Figure 4:**
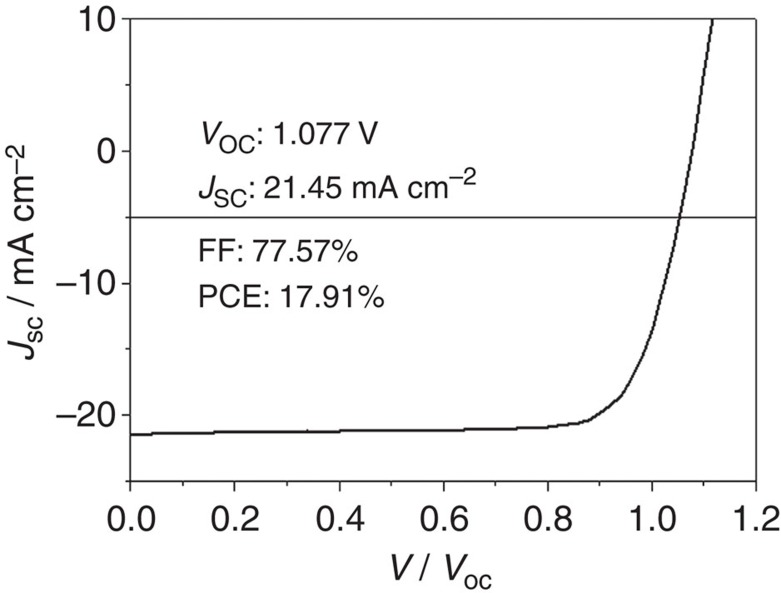
Optimized device performance. Device performance of record solar cell based on Sample 1 with further optimization. The optimized fabrication condition for perovskite solar cell is carried out under the deposition of the mixed solution (CH_3_NH_3_Cl/CH_3_NH_3_I=1:10) on PbI_2_ film, followed by a 135 °C, 15 min baking.

To fully explore the advantages of Cl incorporation on device performance, devices based on Sample 1 were subsequently optimized (Supplementary Figs 3 and 4). First, the annealing time and temperature have been carefully investigated when perovskite crystal growth occurs from *in situ* conversion of PbI_2_ framework. It is found that a relatively higher temperature of 135 °C for a period of 15 min produces the best device performance as a result of the compromise between the grain size and possible defects in the film. The concentration of incorporated chlorine has also been studied to assess its influence on device performance. Measured PCE is maximized when the weight ratio of CH_3_NH_3_Cl/CH_3_NH_3_I used was 1:10, and dropped significantly when the weight ratio was increased to 3:10. Given that the as-prepared film maintains good conformity and surface coverage (Supplementary Fig. 5), this indicates that a high concentration of CH_3_NH_3_Cl is detrimental to device performance, possibly due to increased trap states in the perovskite film or the relevant interfaces. Derived from Sample 1, the best device exhibits outstanding performance with a *J*_SC_ of 21.45 mA cm^−2^, *V*_OC_ of 1.077 V, FF of 77.57% and PCE of 17.91%, as shown in Fig. 4. Studies of device hysteresis and stability are provided in Supplementary Figs 6 and 7, respectively.

## Discussion

**Figure 5:**
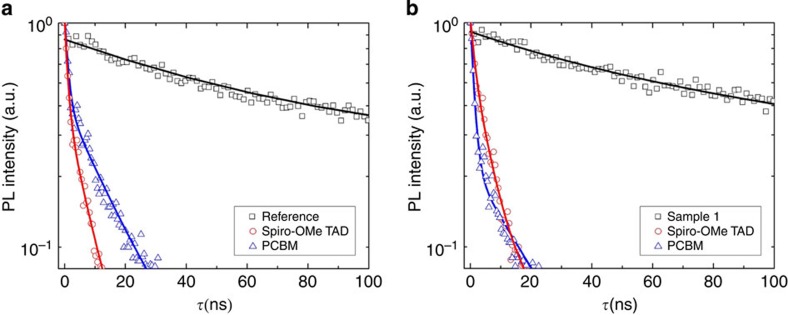
Carrier behaviour of the perovskite film. TRPL measurements and the fitting curves for Reference (**a**) and Sample 1 (**b**) in the presence of quenchers (Spiro-OMeTAD or PCBM, respectively). TRPL measurements taken at the peak emission wavelength are recorded (black square) with an electron transport layer (PCBM; blue triangles) or a hole transport layer (Spiro-OMeTAD; red circles), along with stretched exponential fits in corresponding colours. TRPL spectra were obtained using the time-correlated single-photon counting technique (Picoharp 300) under excitation provided by a picosecond diode laser at a wavelength of 633 nm with a repetition frequency of 1 MHz (PDL 800B).

To gain insight into the effects of Cl incorporation, the carrier transport properties of the Reference and Sample 1 are carefully examined, either within the absorber layer or across the heterojunction. The carrier dynamics in the perovskite heterojunction is reported to follow the relation (1/*τ*_Heterojunction_=1/*τ*_CT_+1/*τ*_Perovskite_), where the two major competing process regarding charge transport are carrier recombination (*τ*_Perovskite_) and charge-carrier transfer (*τ*_CT_)^39^. TRPL measurements are thus employed to extract the carrier lifetime in the perovskite films (*τ*_Perovskite_), which has been successfully applied to describe various radiative and non-radiative loss channels responsible for photoexcited carriers recombination^38^. The samples for this measurement were fabricated on glass substrates in the same condition when the optimized devices were produced. (See Supplementary Information). Figure 5 shows the photoluminescence decay profiles of Sample 1 and Reference with/without quenching layers. Sample 1 (with chlorine inclusion) shows a *τ* value of 241 ns, which is in good agreement to that of CH_3_NH_3_PbI_*3−x*_Cl_*x*_ films by one-step deposition in the absence of quenching layers^38^. Interestingly, a *τ* value of 289 ns was measured for Reference (CH_3_NH_3_PbI_3_) without quenching layer, which was over one order of magnitude higher than that of CH_3_NH_3_PbI_3_ fabricated via conventional one-step deposition as previously reported^38^,^39^. The addition of the PCBM or Spiro-OMeTAD quenching layers accelerates the PL decay for both Reference and Sample 1. The observed time constants for PCBM-quenched Reference and Sample 1 are ∼11.8 and 9.6 ns, respectively, and those for Spiro-OMeTAD quenched ones are 9.6 and 5.6 ns, respectively. Taking into account the comparable carrier diffusion coefficient as previously reported^38^, the corresponding diffusion length for electrons and holes were estimated to well exceed the thickness of the absorber layer for both Reference and Sample 1. These results serve as additional evidence to explain the superior performance of devices in CH_3_NH_3_PbI_3_-based planar configuration documented recently^41^,^42^,^43^. It also suggests that carrier dynamics in perovksite films closely correlates to processing conditions. The exceptionally long carrier lifetime of the Reference agrees with the improved film morphology in terms of both film conformity and grain size while providing an ideal starting point to further unravel material interaction at the interfaces induced by incorporation of extrinsic ions. As the characteristic charge-carrier transfer lifetime *τ*_CT_ is <1 ns (ref. 39), it is reasonable to conclude that 1/*τ*_CT_ is predominant to determine the carrier dynamics across the heterojunction. It implies that carrier lifetimes (*τ*_Perovskite_) with this same magnitude of several hundred ns do not contribute significant variation to the device performance. The comparable carrier lifetimes in both samples indicate that the chlorine incorporation does not affect the non-radiative recombination channels in the perovskite films.

**Figure 6:**
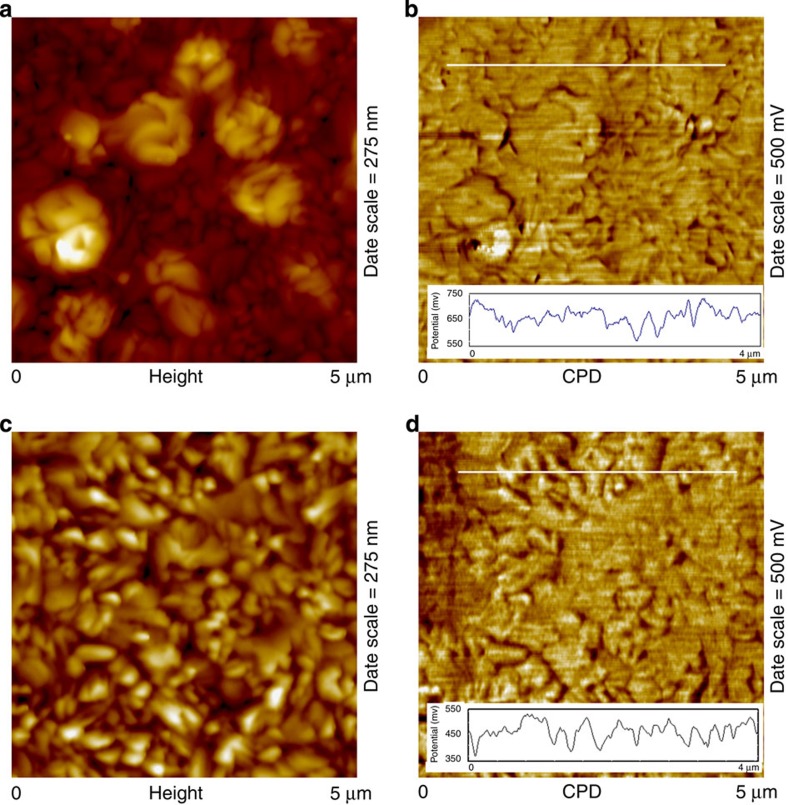
KPFM results of perovskite films. Representative atomic force microscopy height images of (**a**) perovskite film of Reference and (**c**) Sample 1. Co-localized KPFM images of surface work function of (**b**) of Reference and (**d**) Sample 1. Cross-sectional analyses of KPFM data (insets) show variations in local CPD of up to ±160 mV in magnitude across the sample surface with minimal variation (±20–40 mV) at GBs.

The properties of GBs are investigated to further clarify the nature of carrier dynamics within perovskite films on Cl incorporation. Variations in electronic properties at GBs have been reported to effectively boost device performance in conventional as well as perovskite solar cells^44^. Observed energy band bending at GBs is generally thought to facilitate charge separation and transport. Meanwhile, prolonged carrier lifetimes are attributed to the successful passivation of surface states^45^. In the current study, the contribution of surface state passivation is ruled out given the similar values of carrier lifetime in both Reference and Sample 1. The local surface potential of each sample has been characterized using KPFM to examine the effects of chlorine incorporation on energy level alignment between the various layers as well as the possibility of band bending along GBs. Spatial maps of surface topography and corresponding local surface potential for both the Reference and Sample 1 on ITO substrates are shown in Fig. 6. KPFM provides a reliable measurement of local surface potentials stemming from contact potential differences (CPD) between the tip and sample surface associated with their relative work functions. Minimal (±20–40 mV) variations in the CPD were observed between the GB and grain bulk for both samples thereby indicating that chlorine incorporation has a negligible effect on the energy band edge at GBs and thus carrier transport within the perovskite film. In addition, the magnitude of local variations in the CPD (±120–160 mV) were similar for both samples. These grain-to-grain variations appear to depend on the physical orientation of each grain and its associated crystallographic face^43^. Moreover, chlorine incorporation was seen to affect the mean values of the CPD in the perovskite film, which decreased from ∼0.65 to 0.45 V (Fig. 6b,d). The measured CPD of TiO_2_ films (0.4 V), and observed CPD shift towards that of TiO_2_ in perovskite films following chlorine inclusion indicates better energy band alignment, which is likely to facilitate the electron extraction along the interface. Cross-sectional KPFM was used to further assess the energy level band alignment between the absorber and transport layers (Supplementary Fig. 8). A downward shift in CPD similar to that measured at the surface of the perovskite material was confirmed for Sample 1 compared with the Reference, as seen in the corresponding line profiles (Supplementary Fig. 8c,f) and image histograms (Supplementary Fig. 9). An analogous shift in CPD of the Spiro-OMeTAD films deposited on Reference and Sample 1, respectively, was observed (Supplementary Fig. 9). The relatively larger CPD of Spiro-OMeTAD on Sample 1 indicates a deeper Fermi energy level of the hole transport materials serves to enlarge the build-in potential and improve device performance through an increase in *V*_OC_. In this regard, it is inferred that the Cl incorporation to improve optoelectronic properties of perovskite devices may associate with both interfaces.

**Figure 7:**
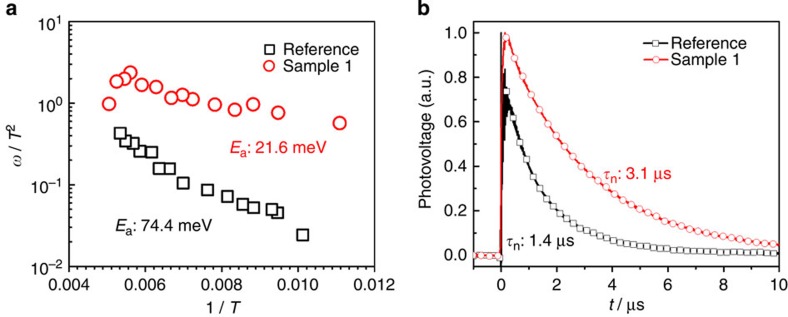
Defect and carrier behaviour of the perovskite devices. (**a**) Arrhenius plots of the inflection frequencies in the admittance spectra for devices based on Reference (squares) and Sample 1 (circles) determined from the derivative *f*d*C/*d*f*. The value *E*_a_ is the activation energy for the traps, where *E*_a_ for Reference and Sample 1 is 74.4 and 21.6 meV, respectively. (**b**) Transient photovoltage decay curves and extracted lifetime of the injected carrier in the devices based on Reference (squares) and Sample 1 (circles). The charge lifetime in Sample 1 with chlorine incorporation was measured to be two times longer than that of Reference.

We further investigate carrier recombination dynamics in the scope of the entire heterojunction across the device. Transient photovoltage decay was perfomed under open circuit condition with one sun illumination (See Supplementary Information). In this scenario, the carriers cannot be swiped out of the device but recombine, so transient photovoltage decay correlates to carrier recombination lifetimes in the working device^46^. The photovoltage decay curves for devices based on Sample 1 and Reference are plotted in Fig. 7. In both cases, the photovoltage decay constants are on the scale of microseconds, in accordance to the recent report^9^. The charge lifetime in the device with chlorine incorporation was measured to be two times longer than that of Reference. This indicates that carrier recombination along the entire device has been suppressed due to chlorine incorporation, which consequently improves the device efficiency. Since the perovskite film itself does not exhibit convincing evidence for significant reduction of the carrier recombination neither in the bulk nor along the GBs, it is expected that the improvement possibly originates from the interfaces between the perovskites and the relevant carrier transport layers due to the chlorine inclusion.

CV measurements are frequently employed to probe the carrier behaviour within PV devices, and the activation energy of defects in the materials are able to be extracted consequently^47^. Carrier recombination dynamics are dependent on the defect activation energy in the absorber. Using the CV technique, the defect activation energy of Sample 1- and Reference-based devices are calculated to be 21.6 and 74.4 meV, respectively (Fig. 7). It means chlorine incorporation during perovskite film growth clearly leads to shallower defect along the entire working device. Generally, these shallow defects do not serve as recombination centres in the bulk to affect its carrier lifetime, as observed in our TRPL measurement (Fig. 5). In addition, careful KPFM examination has excluded the possibility that chlorine influences the GBs (Fig. 6). It thus indicates that the shallower defects might originate from the more energetically favourable interface between perovskite/carrier transport layer, and hence result in an improved carrier extraction, which is ultimately responsible for the enhancement of FF and *V*_OC_ in the device (Fig. 3). These results are in good agreement with transient photovoltage measurements showing the carrier recombination rate to be lower in chlorine-incorporated devices. Given the negligible morphological improvement as shown in SEM and atomic force microscopy, it could be further deduced that the improved interface is not solely from better physical contact to carrier transport materials. The improvement in device performance is likely due to the synergetic effect of energy level alignment at the improved interfaces in contact with carrier transport materials by chlorine inclusion. Our results are in accordance with recent studies based on angle-resolved XPS and first-principles density functional theory modelling^48^. These XPS results showed direct evidence that chloride is preferentially located in close proximity to the perovskite/TiO_2_ interface, while simulation results indicated the establishment of a directional ‘electron funnel' that may improve the charge collection efficiency of the device^48^. Further investigation on the carrier dynamics along each relevant interface is currently underway.

In summary, we report a rational investigation of the Cl effect on perovskite materials properties through decoupling the most influential morphological factors that affect device performance significantly. Conceptually different from previous work, the present CH_3_NH_3_PbI_3_ and CH_3_NH_3_PbI_3_(Cl) films show consistent conformity, which alleviates morphological difference by chlorine incorporation. XRD and XPS characterization excludes the Cl effect on the crystal structure and composition change of the perovskite film. Specific to the current approach, Cl incorporation does not affect carrier transport characteristics within the perovskite film but still improves the device performance. Transient photovoltage decay, CV characterization and cross-sectional KPFM, based on measuring the entire perovskite heterojunction, confirm the improved interface between perovskites and carrier transport layers. This investigation approach presents a simple, reliable and versatile approach towards examining the origin of extrinsic ion effects and high performance in perovskite PVs. Besides Cl, other extrinsic species can be readily incorporated into the system by simply switching the reactants. Continuous efforts will be focused on the investigation of charge transfer behaviour across the interfaces to further boost the device performance. It can be expected that with further exploration of the chemical and physical properties of crystal structures, perovskite solar cells will ultimately offer an excellent mix of performance, material abundance and scalable fabrication, potentially becoming the prevailing thin-film technology in next-generation PV devices.

## Methods

### Device fabrication

Methylammonium iodide (CH_3_NH_3_I, MAI) and methylammonium chloride (CH_3_NH_3_Cl, MACl) were synthesized using the method described elsewhere^9^. The TiO_2_ nanocrystals were obtained from a non-hydrolytic sol-gel approach^9^. ITO or glass substrates (for PL measurements) were sequentially washed with isopropanol, acetone, distilled water and ethanol. The ETL was subsequently coated on ITO substrates with a TiAcac-stabilized TiO_2_ solution and annealed at 150 °C for 30 min in air. PbI_2_ (dissolved in dimethylformamide, 450 mg ml^−1^) was spin-coated on top of ITO/TiO_2_ substrate at 2,500 r.p.m. for 30 s. Then MAI (dissolved in 2-propanol) or a mixture of MAI/MACl was spin-coated on top of the dried PbI_2_ layer at room temperature at 3,000 r.p.m. for 30 s in the dry air (at a dew point of −70 °C). All of the films were annealed in the air at 135 °C for desired time. A hole transport layer solution was coated on the perovskite film at 3,000 r.p.m. for 30 s, where a spiro-OMeTAD/chlorobenzene (90 mg per 1 ml) solution was employed with addition of 45 μl Li-TFSI/acetonitrile (170 mg per 1 ml) and 10 μl tBP. Finally, the counter electrode was deposited by thermal evaporation of gold under a pressure of 5 × 10^−5^ Torr. The active area was 0.108 cm^2^. The as-fabricated device depicts from the bottom to the top, the 150 nm thick ITO electrode, 40 nm of Y-TiO_2_, 350 nm of perovskite, 200 nm of spiro-OMeTAD and 100 nm of gold. The details for characterization are described in Supplementary Information.

## Additional information

**How to cite this article:** Chen, Q. *et al*. The optoelectronic role of chlorine in CH_3_NH_3_PbI_3_(Cl)-based perovskite solar cells. *Nat. Commun*. 6:7269 doi: 10.1038/ncomms8269 (2015).

## Supplementary Material

Supplementary Information Supplementary Figures 1-9, Supplementary Table 1 and Supplementary Methods

## Figures and Tables

**Table 1:** Summary of the average photovoltaic parameters of devices based on Reference and Samples 1 and 2.

	*V*_oc_ (V)	*J*_sc_ (mA cm^−2^)	FF (%)	PCE (%)
Reference	1.003	19.58	71.31	14.12
Sample 1	1.029	21.08	74.52	16.11
Sample 2	0.999	20.1	70.26	14.22

FF, fill factor; PCE, power conversion efficiency.
