# Supplementary material for: The optoelectronic role of chlorine in CH3NH3PbI3(Cl)-based perovskite solar cells
Source: Nat Commun. 2015 Jun 12;6:7269. doi: 10.1038/ncomms8269 (PMC4490385; doi:10.1038/ncomms8269)
Supplement: Supplementary Information — Supplementary Figures 1-9, Supplementary Table 1 and Supplementary Methods [file ncomms8269-s1.pdf]

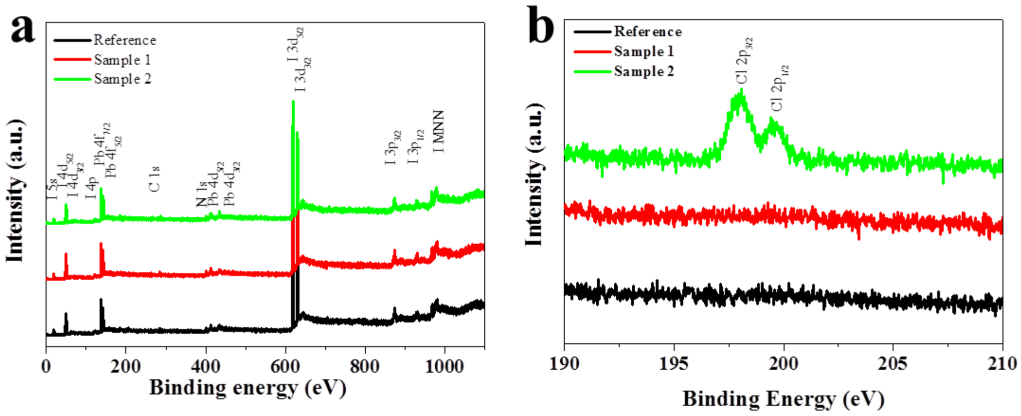

Supplementary Figure 1 XPS characterization of perovskite film. (a) XPS survey spectrum of the top surface of the perovskite films of Reference, Sample 1&2; **(b)** Cl2p core level XPS spectra of the perovskite films, characteristic peak binding energy of Cl2p 3/2 and Cl2p 1/2 (198.9 eV and 200.5 eV) are indicated.

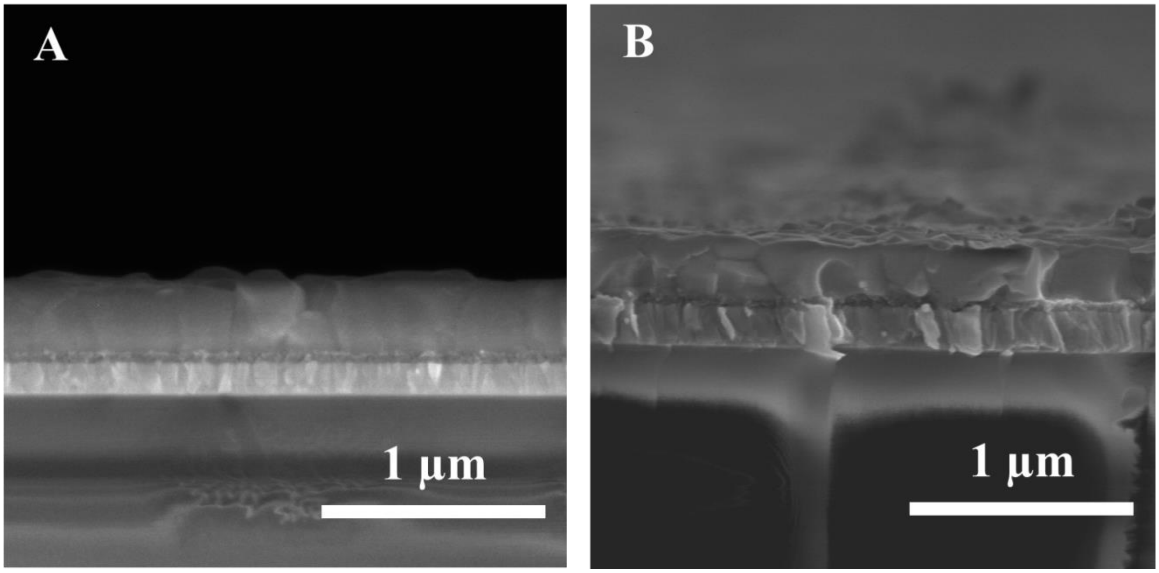

Supplementary Figure 2 SEM images of perovskite film. The cross-sectional SEM images of Sample 1 (A) and Reference (B) fabricated on the ITO/TiO_2 substrates.

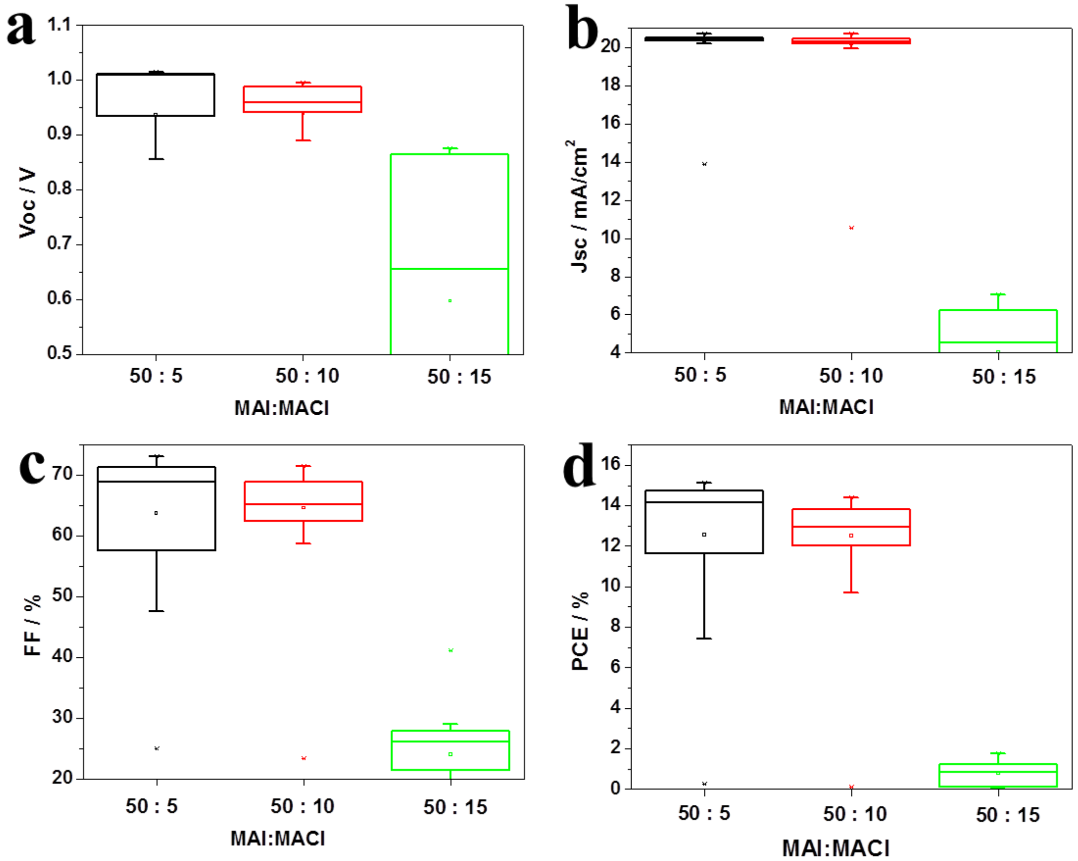

Supplementary Figure 3 Device performance of different perovskite cells. Photovoltaic parameters: V_{OC} **(a)**, J_{SC} **(b)**, FF **(c)**, and PCE **(d)**, extracted from current-voltage measurements of a series of solar cells via Sample 1 protocol with different concentration of mixture of MAI/MACl (in weight ratio).

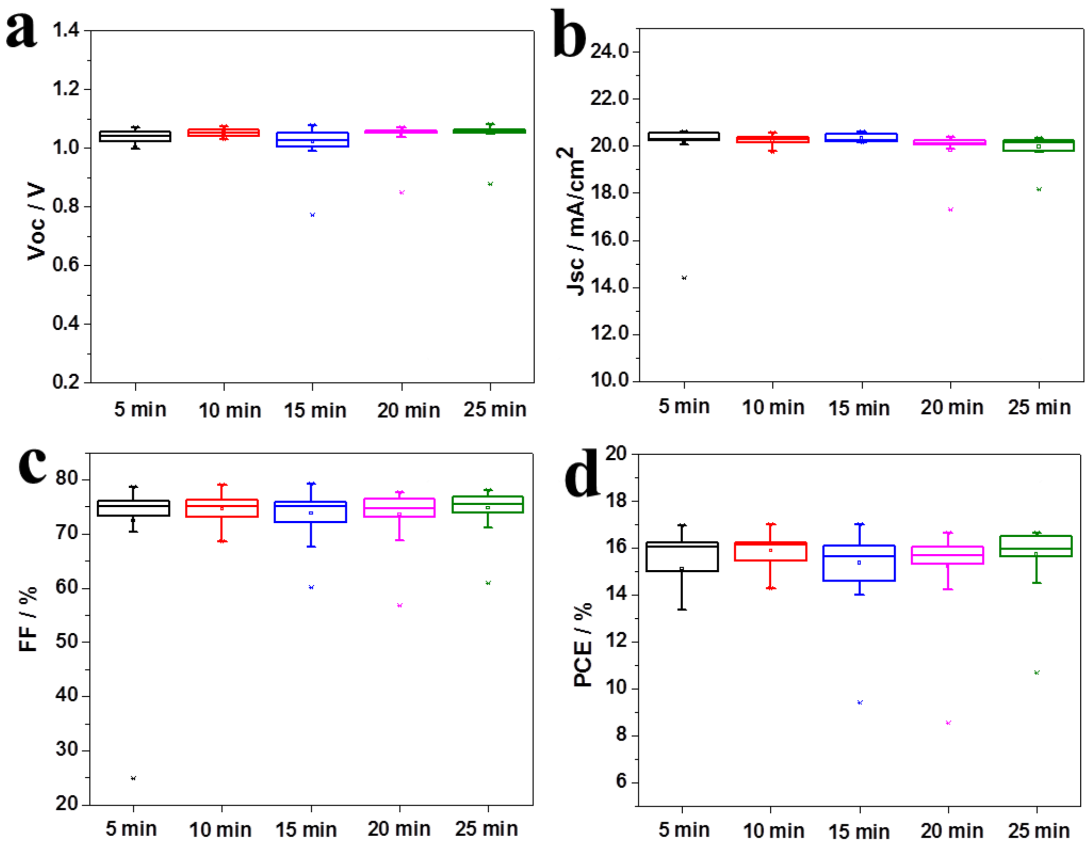

Supplementary Figure 4 Device performance of perovskite cells based on different fabrication time. Photovoltaic parameters: V_{OC} **(a)**, J_{SC} **(b)**, FF **(c)**, and PCE **(d)**, extracted from current-voltage measurements of a series of solar cells via Sample 1 protocol with different annealing time.

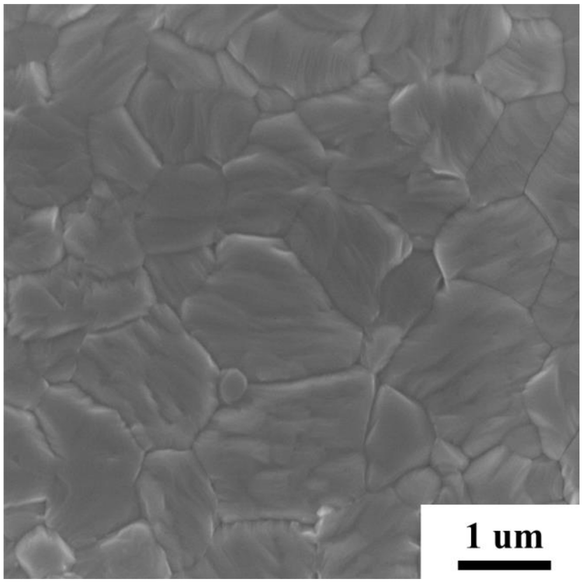

Supplementary Figure 5 SEM image of perovskite film. Top-view SEM image of perovskite films on the TiO_2 substrates fabricated from the precursors mixture of CH_3NH_3Cl and CH_3NH_3I (3:10 in weight).

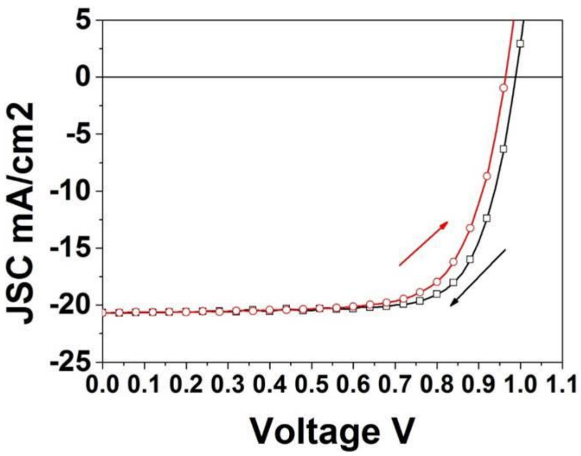

Supplementary Figure 6 Hysteresis behavior of perovskite solar cell. Current-voltage curves of a typical device based on Sample 1 under AM 1.5 simulated illumination. From forward bias to short-circuit (red circles) (1.2 V→ 0 V) and from short-circuit to forward bias (black squares) (0 V→1.2 V) current density-voltage curves were obtained from different sweep directions. The step size is 20 mV.

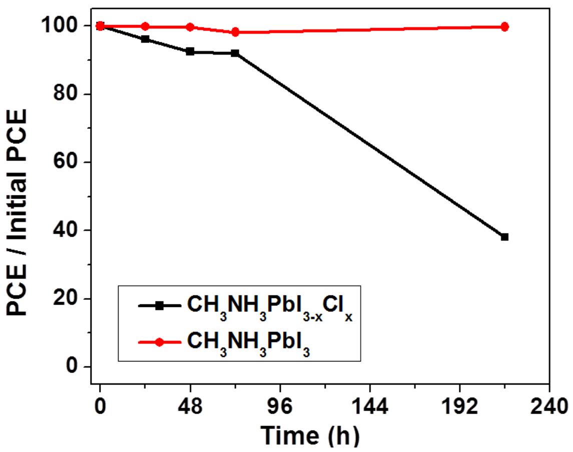

Supplementary Figure 7 Stability of perovskite solar cell. Stability investigation on the perovskite solar cells based on Reference (red circles) and Sample 1 (black squares) The stability test in each condition is based on over 10 devices. The test was on the devices stored in dry air without encapsulation.

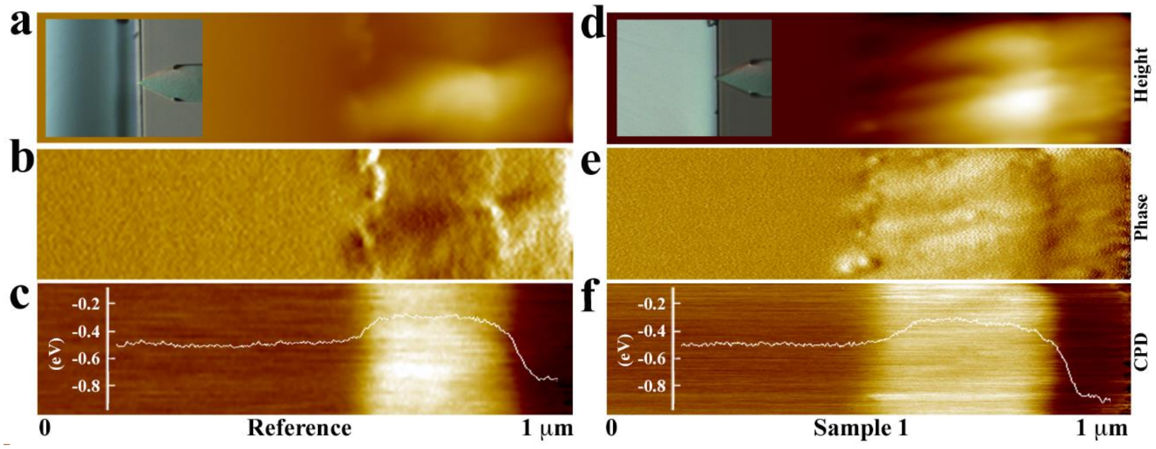

Supplementary Figure 8 KPFM characterization of perovskite solar cell. Simultaneously acquired height (a,d), phase (b,e) and surface potential (c,f) images of the Reference (a-c) and Sample 1 (d-f) cross-section acquired by scanning the KPFM tip (OSCM-Pt) across a freshly cleaved Si-TiO_2-perovskite- spiro-OMeTAD interface (insets).

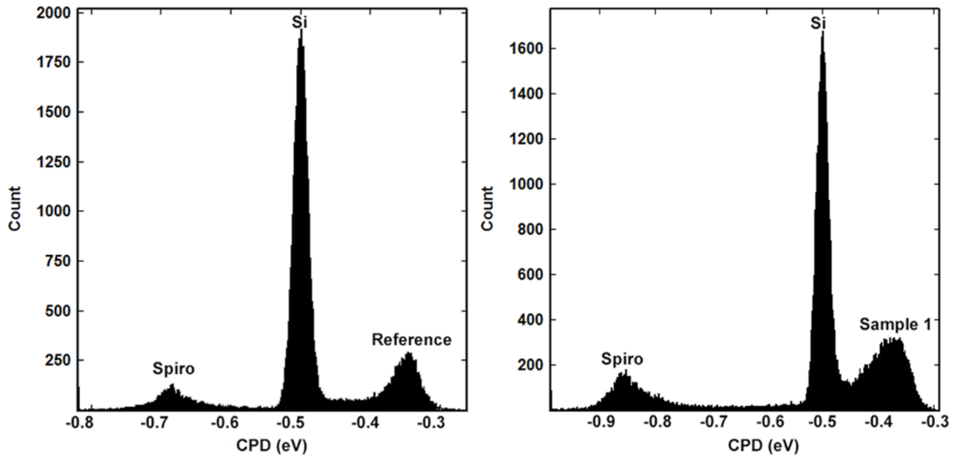

Supplementary Figure 9 KPFM analysis of perovskite solar cell. Image histogram of surface CPDs from cross-sectional KPFM analysis of Figure S8 (acquired with OSCM-PT) tip showing clearly defined peaks corresponding to each layer of the structure. A shift in the mean CPD values for both the perovskite and spiro-OMeTAD layers are clearly observed following Cl incorporation.

Supplementary Table 1 Hysteresis behavior of perovskite solar cell.

	**Voc (V)**	**Jsc (mA/cm^2)**	**PCE (%)**	**FF (%)**
Reverse Fast	0.988	20.66	15.30	74.94
Forward Fast	0.964	20.69	14.40	72.23

Supplementary Methods:

All the chemicals were used as received, including PbI_2 (99.999%, Sigma-Aldrich), HI (57 wt% in water, Aldrich), HCl, CH_3NH_2 (33 wt. % in absolute ethanol, Sigma-Aldrich), $TiCl_4$, Titanium diisopropoxide bis(acetylacetonate) 75% in isopropanol (TiAcac, Sigma-Aldrich), spiro-OMeTAD (Lumtec), dimethylformamide (DMF), Li-bis(trifluoromethanesulfonyl) imide (Li-

TFSI, Sigma), Polyethylenimine, 80% ethoxylated solution 35-40 wt. % in H_2O (Sigma), diethyl ether, acetone, isopropanol, acetonitrile, ethanol, 2-methoxyethanol, benzyl alcohol. Fullerene [phenyl-C61-butyric acid methyl ester (PCBM)] was obtained from Nano-C.

Device Fabrication: Methylammonium iodide (CH_3NH_3I, MAI) and Methylammonium Chloride (CH_3NH_3Cl, MACl) were synthesized using the method described elsewhere (Refers to *Science*, 2014, 345, 542-546). The TiO_2 nanocrystals were obtained from a non-hydrolytic sol-gel approach (Refers to *Science*, 2014, 345, 542-546). ITO or glass substrates (for PL measurements) were sequentially washed with isopropanol, acetone, distilled water and ethanol. The ETL was subsequently coated on ITO substrates with a TiAcac stabilized TiO_2 solution and annealed at 150 °C for 30 min in air. PbI_2 (dissolved in DMF, 450 mg/ml) was spin-coated on top of ITO/TiO_2 substrate at 2500 rpm for 30 s. Then MAI (dissolved in 2-propanol) or a mixture of MAI/MACl was spin-coated on top of the dried PbI_2 layer at room temperature at 3000 rpm for 30 s in the dry air (at Dew Point of -70 °C). All of the films were annealed in the air at 135 °C for desired time. A hole transport layer (HTL) solution was coated on the perovskite film at 3000 rpm for 30s, where a spiro-OMeTAD/chlorobenzene (90 mg/1 mL) solution was employed with addition of 45 µL Li-TFSI/acetonitrile (170 mg/ 1 mL) and 10 µL tBP. Finally, the counter electrode was deposited by thermal evaporation of gold under a pressure of 5×10^{-5} Torr. The active area was 0.108 cm^2. The as-fabricated device depicts from the bottom to the top, the 150 nm thick ITO electrode, 40 nm of Y-TiO_2, 350 nm of perovskite, 200 nm of spiro-OMeTAD, and 100 nm of gold.

Characterizations: X-ray diffraction patterns (2θ scans) were obtained from samples of perovskite deposited on the FTO/c-TiO_2 substrates a double axis X-ray diffractometer (XRD, Bede D1 diffractometer equipped with a focusing graded X-ray mirror with monochromatic CuKα (λ = 1.5405 Å) radiation source. Scans were taken with 0.5 mm wide source and detector slits, a step size of 0.025 °, and a counting time of 2.5 s with generator settings at 40 kV and 30

mA. A field emission SEM (FEI Nova 230 NanoSEM) was used for acquiring SEM images. The instrument uses an electron beam accelerated at 500V to 30 kV, enabling operation at a variety of currents. Scanning Kelvin Probe Force Microscopy was performed on perovskite samples in ambient conditions using a Dimension Icon Scanning Probe Microscope (Bruker Nano, Inc.) in a single-pass frequency modulated (FM-KPFM) mode. FM detection maximizes spatial resolution by measuring the local electrostatic force gradient present solely at the apex of the AFM tip, thereby reducing contributions of the tip cone and cantilever present in more common amplitude modulated detection schemes. Application of an off-resonant AC voltage (∼5 V, 2 kHz) between a conductive AFM probe (OSCM-Pt or PFQNE-AL, Bruker) and the grounded sample alters the effective spring constant of the cantilever and thus modulates its resonant frequency due to an induced electric force gradient. Spatial variations in the surface potential/work function were directly measured by nulling local electrostatic force gradient arising from contact potential differences between the AFM tip and the device surface through use of a bias-controlled feedback loop. A minimum force setpoint was employed, as indicated by nominal phase contrast, in order to reduce potential artifacts in the surface potential images. Calibration of the tip (PFQNE-Al) work function, using a freshly cleaved HOPG substrate of known work function (4.6 eV), enabled reporting of surface work functions for perovskite samples (Figure 5 and S9). Positioning the tip (OSCM-Pt) on the face of a freshly cleaved device enabled acquisition of cross-sectional FM-KPFM images (Figure S8 and S10) across the Si-TiO_2-perovskite-spiro interface. Current density (J)-voltage(V) curves were measured using a Keithley 2401 source-measure unit under AM1.5G illumination at 100 mW/cm^2 provided by a Oriel Sol3A solar simulator. Light intensity was determined by KG-5 filter diodes as a reference cell. The J-V curves were obtained in air through reverse (1.2 V→ 0 V) or forward (0 V→1.2 V) scans with the step size of 20 mV. The masking active area is 0.108 cm^2. Differential light intensity response measurements utilized neutral density filters and aperture sizes to tune the light intensity, and a Si-diode was used to calibrate the light intensity. Steady-state photoluminescence (PL) was analyzed using Horiba Jobin Yvon

system with an excitation at 640 nm. Time-resolved photoluminescence spectra were obtained using the time-correlated single-photon counting technique (Picoharp 300) under excitation provided by a picosecond diode laser at a wavelength of 640 nm with a repetition frequency of 1 MHz (PDL 800B). For transient photovoltage decay measurements, a white light bias was generated from an array of diodes (Molex 180081-4320) to simulate 1 sun working condition. A pulsed red dye laser (Rhodamine 6G, 590nm) pumped by a nitrogen laser (LSI VSL-337ND-S) was used as the perturbation source, with a pulse width of 4 ns and a repetition frequency of 10 Hz. The perturbation light intensity was attenuated to keep the amplitude of transient Voc (ΔVoc) below 5 mV so that ΔVoc $\ll$ Voc. Voltage dynamics were recorded on a digital oscilloscope (Tektronix DPO 4104B), and voltages at open circuit were measured over a MΩ and a 50Ω resistor, respectively. Admittance spectroscopy was conducted using a Hewlett-Packard 4284A LCR Meter. Electrical contact to the devices during LCR measurements was provided by a Janis cryogenic probe station with anattached Lakeshore 331 temperature controller where the temperature was monitored using a thermocouple placed in contact with the sample location.
